# A Single Preoperative Dose of Pregabalin and Chronic Postsurgical Pain Following Elective Coronary Artery Bypass Graft Surgery: A Secondary Analysis from a Randomized, Double-Blind, Placebo-Controlled Trial

**DOI:** 10.3390/jcm15041648

**Published:** 2026-02-22

**Authors:** Aikaterini Bouzia, Konstantinos Tassoudis, Vasilis Tassoudis, Maria P. Ntalouka, Anastasia Michou, Metaxia Bareka, Eleni Arnaoutoglou

**Affiliations:** Department of Anesthesiology, Faculty of Medicine, School of Health Sciences, University of Thessaly, University Hospital of Larissa, 41110 Larisa, Greece; catbouzia@yahoo.gr (A.B.); kostastass98@gmail.com (K.T.); barekametaxia@hotmail.com (M.B.)

**Keywords:** cardiac anesthesia, cardiac surgery, pregabalin, postoperative pain, chronic pain

## Abstract

**Background/Objectives**: Chronic persistent postoperative pain after cardiac surgery, first described as Post CABG Pain Syndrome (PCPS), has been a well-recognized problem since 1989. This study investigated the effect of a single preoperatively administrated pregabalin dose on chronic persistent postoperative pain, in terms of pain intensity, rescue analgesia, and sleep disturbances, after elective cardiac surgery. **Methods**: This is a secondary analysis of a prospective double-blind single center study that took place in a tertiary/referral center (NCT01701921). Consecutive adult patients who underwent elective cardiac surgery with median sternotomy and extracorporeal circulation under general anesthesia were included. Patients were randomly assigned into three groups {placebo (group 1), oral pregabalin 75 mg (group 2), oral pregabalin 150 mg (group 3)}. Placebo or either dose of pregabalin were administered 1 h preoperatively. Postoperatively at 12 and 24 months, the presence of persistent chronic pain (Numeric Rating Scale, NRS), the need of daily intake of analgesics, and any potential sleep disturbances were assessed. **Results**: In total, 93 out of 108 patients completed this secondary analysis (86,1%). Patients from all three groups reported persistent postoperative pain at 12 and 24 months, respectively. The mean NRS scores at 12 months were 1.71 (0.588) group 1, 1.23 (0.560) group 2, and 1.19 (0.402) group 3. At 24 months, the mean NRS scores were 1.19 (0.543) Group 1, 0.84 (0.454) Group 2, and 0.84 (0.374) Group 3. No statistically important difference was detected between the two different pregabalin groups. Regarding the use of analgesics, Pearson Chi-Square showed *p*-values *p* = 0.027 in 12 months and *p* = 0.01 in 24 months and lower scores were detected in the high pregabalin dose group. As far as the sleep disturbances are concerned, there was no significant difference between groups. The number of patients who reported persistent postoperative pain at 12 and 24 months was significantly lower in the pregabalin groups (75 mg and 150 mg) than in the placebo group. Regarding the NRS score, Kruskal–Wallis showed a value of *p* = 0.001 and *p* = 0.005 in 12 and 24 months respectively. Regarding the use of analgesics, Pearson Chi-Square showed *p*-values *p* = 0.027 in 12 months and *p* = 0.01 in 24 months. Referring to sleep disturbances, there was no significant difference between groups. **Conclusions**: Based on the results of this study, it seems that oral administration of a single preoperative dose of pregabalin may exhibit a significant preventive effect on chronic persistent postoperative pain after elective cardiac surgery.

## 1. Introduction

Recent studies show that the number of surgeries taking place across Europe is over 50 million per year [1]. The increasing number of all types of surgical procedures performed every year has raised concern about the effect of postsurgical pain on patient quality of life and the associated economic burden. Postsurgical pain should normally resolve after 1–2 weeks depending on the type of surgery, after tissue healing is complete. Unfortunately, that is not always the actual course of events [2]. A significant number of patients will experience chronic or persistent postsurgical pain (CPSP), lasting long beyond the expected healing period. CPSP after non-cardiac surgery and cardiac surgery is often underdiagnosed or underestimated, and many patients remain untreated. For that reason, since 2019, the International Association of the Study of Pain (IASP) has redefined CPSP as pain that develops or increases in intensity after a surgical procedure, persists for at least 3 months, and is localized to the surgical field [3].

To minimize the adverse effects of persistent postsurgical pain, many studies investigate the risk factors for developing CPSP and the means of preventing the transition from acute to chronic pain [4]. Although the precise mechanisms driving the transition from acute to chronic or persistent postsurgical pain remain unclarified, overwhelming evidence suggests that this process is modulated by a complex interplay of biological, psychological, and socioeconomic factors [5]. Among the factors that contribute to the development of CPSP are preexisting pain, genetics, psychological factors, intensity of acute postoperative pain, and nerve injury during the surgery [6]. A study conducted by Sun, Terri et al. found that a higher pain score on the first postoperative day, history of chronic pain prior to surgery, and history of depression were major risk factors [7]. Many researchers, such as Richebé et al. [6] and Gjeilo, Kari Hanne et al. [8], emphasize the need for preventive measures to avoid poorly treated acute postoperative pain. For many years, multimodal analgesia has been regarded as providing a more effective way to control acute postoperative pain, and in that context, many medications have been tested for their analgesic and preventive effects perioperatively [9,10].

This secondary analysis of a prospective double-blind, placebo controlled, single center study aimed to investigate the effect of a single preoperatively administrated pregabalin dose on chronic persistent postoperative pain at 12 and 24 months, in terms of pain intensity, rescue analgesia, and sleep disturbances, after elective cardiac surgery. In addition, we evaluated the possible effect of two different (75 mg versus 150 mg) pregabalin doses in terms of pain intensity, rescue analgesia, and sleep disturbances at 12 and 24 months, respectively. Sources of pain after cardiac surgery with median sternotomy include sternotomy incision, chest tubes, pericardial drainage, the site of saphenous vein, or radial artery harvesting. The pain is described as chest discomfort of noncardiac origin in up to 65% of cases and sometimes coexists with pain in the upper extremities, neck, head, and midback area. The reported pain by patients is mixed.

## 2. Materials and Methods

### 2.1. Study Cohort

This is a secondary analysis of a prospective double-blind, placebo controlled, randomized study which was conducted in a tertiary/referral center (General University Hospital of Larissa, Greece) [11]. Patients were assessed at 12 and 24 months postoperatively after informed consent was obtained, according to the Helsinki Declaration on patient safety during anesthesiology research. At the outset, after approval by the Ethics Committee, adult patients who underwent elective cardiac surgery with median sternotomy and extracorporeal circulation under general anesthesia were invited to participate. The study protocol was registered in the “ClinicalTrials.gov” clinical trial registration website (ClinicalTrials.gov identifier: NCT01701921).

#### 2.1.1. Inclusion and Exclusion Criteria

Inclusion criteria were elective primary cardiac surgery with median sternotomy and extracorporeal circulation, ages 18–85 years, and written informed consent for participation in the study. Any patient with history of cardiac or thoracic surgery, allergy to pregabalin, previous exposure to gabapentin or pregabalin, preexisting chronic pain, history of depression or other psychiatric diseases, cognitive impairment or inability to be interviewed for the study, renal insufficiency, and history of substance abuse was excluded.

#### 2.1.2. Randomization

Patients were randomly assigned, according to a computer custom number generator, to one of three following groups [Group 1 = control (placebo), Group 2 = 75 mg dose of pregabalin, and Group 3 = 150 mg dose of pregabalin]. All medication, pregabalin and/or placebo, were administered orally, one hour prior to surgery with the use of a “blind method”.

The primary outcome of this study was to detect any difference in pain scores during the first postoperative day after extubation and the onset of CPSP between patients of all the above groups from 3 until 24 months after surgery, essentially exploring if pregabalin could have a positive effect on pain.

In the first part of our study, we looked at the effect on morphine consumption over the first 24 h after surgery, chronic pain 3 months after the surgery, analgesics consumption, and sleep disturbances [11]. The results were encouraging, in that preoperative pregabalin had a beneficial effect on acute postsurgical pain and chronic pain at 3 months. In the later part of the study, we aimed at investigating the impact of this single preoperative oral dose of pregabalin on the transition from acute to chronic pain up to 24 months in patients undergoing elective cardiac surgery with median sternotomy.

#### 2.1.3. Perioperative Care

All patients participating in the study underwent cardiac surgery (CABG) performed by the same surgeon, under general anesthesia, with median sternotomy and extra-corporeal circulation, and received the same type of anesthetic and analgesic medication perioperatively, in similar doses according to safety protocols. All were transferred to the ICU after the surgery, where they were extubated and received the same kind of analgesics postoperatively. Intravenous morphine with patient-controlled analgesia (PCA) was initiated with a programmable PCA pump. Full details of the above mentioned, as the “blind method”, patient management during surgery, postoperative pain relief, and supplemental analgesia, as other data or tables as a consort diagram, are presented and explained in the first published part of this work [11].

The second part focusses on patients who were assessed as outpatients at intervals of 12 and 24 months after the surgery, using the same methods (NRS, verbal Numeric Rating Scale, 0 = no pain to 10 = unbearable pain) by an interviewer unaware of the preoperative medication received by each patient. During follow-up interviews, the patients were asked about the presence of pain related to surgery and the intensity of pain. Besides reported pain, patients were also interviewed about the use of analgesic drugs (NO/YES answer) and the presence of sleep disturbances (NO/YES answer).

### 2.2. Statistical Analysis

After collecting the data concerning NRS, the use of analgesic drugs, and the patients who exhibit sleep disturbances at 12 and 24 months, statistical analysis was followed.

To determine any statistical differences between groups, the following procedure was implemented: the 3 groups (Placebo vs. Lyrica 75 mg vs. Lyrica 150 mg) were examined using the NRS score, sleep disturbances (yes or no), and the use of analgesics (yes or no) at 12 and 24 months respectively. The Kolmogorov–Smirnov test was used to assess data normality. Since our data were not normal, non-parametric methods were adopted: the Kruskal–Wallis test for continuous (scale) data followed by a Mann–Whitney test as post hoc testing, if a statistical difference was found, to determine which pair of groups had this difference. The Pearson Chi-Square test was used for qualitative or binary data (analgesic drugs consumption and sleep disturbances); if a difference was found, the groups were compared in pairs to detect that difference, while the Bonferroni correction was applied to avoid possible “significant *p*-values” due to multiple comparisons. Yates’s correction was also used when needed. *p*-values of over <0.05 were considered significant, with IBM SPSS Statistics version: 29.0.2.0 being used for statistical analysis.

## 3. Results

**Figure 1:**
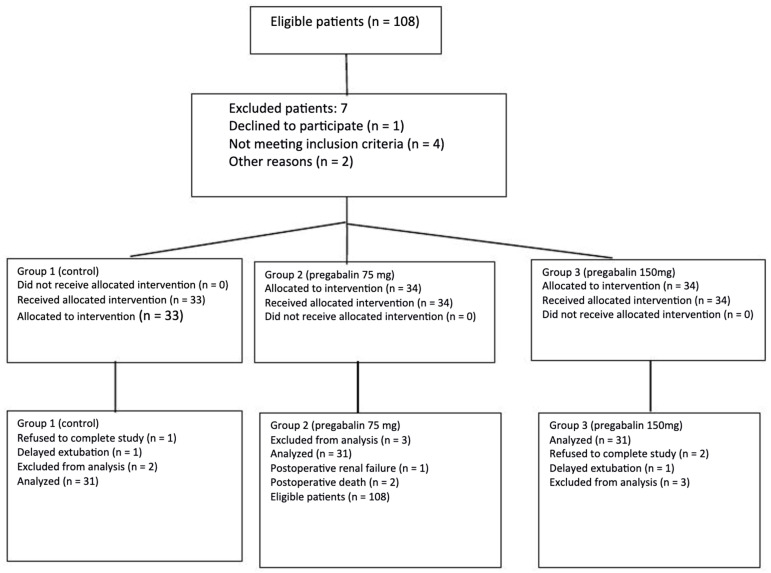
CONSORT diagram showing the progression of patients from eligibility to enrollment to completion of the study.

In total, 93 out of 108 patients completed this secondary analysis (86.1%) (consort flowchart, Figure 1). Demographic and clinical patient characteristics were recorded (Table 1).

This secondary analysis of a prospective double-blind, placebo controlled, single center study aimed to investigate the effect of a single preoperatively administrated pregabalin dose on chronic persistent postoperative pain at 12 and 24 months, in terms of pain intensity, rescue analgesia, and sleep disturbances, after elective cardiac surgery. In addition, we evaluated the possible effect of two different (75 mg versus 150 mg) pregabalin doses in terms of pain intensity, rescue analgesia, and sleep disturbances related to postsurgical pain at 12 and 24 months, respectively. Rescue analgesia included simple analgesics, paracetamol, and NSAIDs.

### 3.1. Outcome in 12 Months

Patients from all three groups reported persistent postoperative pain at 12 months. Mean NRS scores were as follows in each group: 16 patients in group 1 (control), 20 patients in group 2 (Lyrica 75 mg), and six patients in group 3 (Lyrica 150 mg) used rescue analgesia. In contrast, eight patients in group 1, four patients in group 2, and only one patient in group 3 suffered from sleep disturbances. When compared with placebo, patients in the pregabalin groups suffered from lower pain scores (Table 2).

### 3.2. Outcome in 24 Months

A number of patients still reported persistent postoperative pain at 24 months. Mean NRS scores were as follows in each group: nine patients in group 1 (control), three patients in group 2 (Lyrica 75 mg), and one patient in group 3 (Lyrica 150 mg) used rescue analgesia. In contrast, six patients in group 1, two patients in group 2, and only one patient in group 3 suffered from sleep disturbances (Table 2)

The number of patients who reported persistent postoperative pain at 12 and 24 months was significantly lower in the pregabalin groups (75 mg and 150 mg) than in the placebo group. Regarding the NRS score, the Kruskal–Wallis test showed a value of *p* = 0.001 and *p* = 0.005 at 12 and 24 months respectively (Table 2). In the Mann–Whitney test (Table 3), the *p*-value for the placebo vs. Lyrica 75 pair was significant, with a *p* = 0.003 and *p* = 0.008 at 12 and 24 months respectively, as it was in the placebo vs. Lyrica 150 pair (*p* = 0.001 and *p* = 0.005). There was no significant difference in NRS scores between the two Lyrica groups (75 mg and 150 mg) at 12 and 24 months.

Regarding the use of analgesics, the Pearson Chi-Square showed *p*-values of *p* = 0.027 at 12 months and *p* = 0.01 at 24 months. Comparing the three groups in pairs (Table 3), no differences were found, except between the placebo group vs. Lyrica 150 mg group with values of *p* = 0.008 and *p* = 0.006, which became *p* = 0.024 and *p* = 0.018 after the Bonferroni correction at 12 and 24 months respectively. According to Risk Estimate, the odds ratio value was equal to 0.225 for the placebo group vs. Lyrica 150 mg group at 12 months, meaning that a patient from the Lyrica 150 mg group had a 1/0.225 = 4.44 times greater chance of not using analgesics compared to a patient from the placebo group 95% CI (1.42, 13.88). At 24 months, the odds ratio for the same pair was 0.081, meaning that there was a 1/0.081 = 12.34 times greater chance of Lyrica 150 group patients not taking analgesics versus placebo patients at the 95% CI (1.44, 100).

Referring to sleep disturbances, a Pearson Chi-Square showed *p* = 0.037. In the subsequent analysis, the placebo group vs. Lyrica 150 mg group pair had a *p*-value of 0.012. Following Yate’s correction (as more than 20% of cells counted less than five), the new value of *p* was 0.031, and following the Bonferroni correction (multiple comparisons) where 0.05/3 = 0.016 was the new limit, our *p* = 0.031 value was >0.016, indicating no significant difference between groups.

Relatively low NRS scores are expected 12 months and 24 months after the surgery, which is considered enough healing time. By that time, patients should not experience any pain related to the surgery, have sleep disturbances, or take analgesics for persistent postsurgical time. There is clinical significance in those differences between patient groups, as their everyday life can be affected with the administration of pregabalin preoperatively.

## 4. Discussion

Chronic postsurgical pain (CPSP) is a common complication after many surgical procedures, including cardiac surgery. The development of CPSP remains a major health issue for many countries and has a devastating impact on postsurgical rehabilitation and quality of life [1]. To prevent the transition from acute to chronic postoperative pain, a variety of methods and alternative analgesic agents including gabapentinoids have been extensively investigated, as this transition is a complex process [12]. Research in animal models has helped to identify the neurobiological foundation of surgery-induced pain sensitization. Pain after surgery is a result of both inflammation and nerve injury. Cardiopulmonary bypass during CABG is known to induce intense inflammation. The presence of inflammation, which is a substantial contributing factor in pain sensitization, is a key factor in the development of chronic pain. The intensity of pain felt is directly proportional to the intensity of nocipeptive inputs following tissue damage. Nevertheless, it is also affected by central and peripheral mechanisms that enhance the perception of post-incisional pain where nociception produces cellular and molecular alterations [6].

Pregabalin is among those medications that have been investigated for their beneficial effect on postoperative pain [13,14]. Pregabalin binds to the α2-δ protein subunit of presynaptic voltage-gated calcium channels in both the central and peripheral nervous systems, reducing the excitability of nerve synapses and the release of various excitatory neurotransmitters. It has been used for the treatment of neuropathic pain, and many studies suggest that when pregabalin is administered preoperatively, it can contribute to the management of postoperative pain by reducing the occurrence of central sensitization [15]. In a recent study, oral pregabalin during the perioperative period of cardiac surgery was found to significantly reduce 24 h postoperative morphine consumption and shorten the patient’s hospital stay [16].

Cardiac surgery involves invasive procedures such as median sternotomy, retraction of the ribs, harvesting of the internal mammal artery, and leg incisions. Chronic postsurgical pain after cardiac surgery is described by patients as thoracic pain at the sternotomy site and pain at the site of saphenous vein or radial artery after vessel harvesting [17]. The pain may present neuropathic and/or inflammation characteristics. It is very important that differential diagnostic investigation be made to exclude myocardial ischemia, sternal instability, and mediastinitis, since these are common complications after cardiac surgery. Studies have shown that untreated postsurgical pain can still affect patient recovery both 6 and 12 months after cardiac surgery [18]. The severity of that pain has a devastating effect on patients’ quality of life, with one-third of patients reporting sleep disturbances [19]. The prevalence of CPSP after cardiac surgery ranges from 10% to 56% [8] and has been associated with poor functional recovery and the development of psychological effects such as anxiety, fatigue, and depressive disorders [5]. In a study that observed health-related costs after cardiac surgery, it was reported that in patients with CPSP, the financial burden was worse 1 year after the surgery, and that it contributed to work loss due to disability issues and the need for pain-related health care [20].

The results of our study suggest that even a single dose of pregabalin administered orally before cardiac surgery may have a preventive effect on chronic postsurgical pain 12 months and 24 months after the surgery. The use of analgesics for CPSP was also affected in the group of patients who received a higher dose of pregabalin. Given preoperatively, pregabalin has been suggested by numerous studies as playing a role in the management of postoperative pain by reducing the occurrence of central sensitization [21]. It has also been suggested that when analgesics are administered prior to tissue damage, they can reduce the hyperexcitability of dorsal horn neurons and central sensitization [22,23]. Wang, Xian-Xue et al. reported that when administered perioperatively for cardiac surgery patients, oral pregabalin can significantly reduce 24 h postoperative morphine consumption, shorten the patient’s hospital stay, and contribute to early postoperative recovery [16]. In recent studies, different regimens have been investigated, such as single dose pregabalin before surgery, combination or preoperative and postoperative administration of pregabalin, as well as different dosages, ranging from 50 mg up to 300 mg [24].

In our study, we choose to administer a single dose of pregabalin only preoperatively, at two relatively low dosages compared to other studies. Salah Abdelgalil, Ahmed et al. reported that in order to prevent post thoracotomy pain, 300 mg of pregabalin was administered perioperatively. In their research, the authors found that there was a significant increase in the risk of sedation with both single and multiple doses of pregabalin (300 mg), while there was an increase in the risk of severe sedation with multiple doses of 300 mg of pregabalin [25]. It is well known that gabapentinoids are associated with several adverse effects, including sedation, dizziness, and peripheral edema, which are dose-dependent [26]. Administering high doses of pregabalin perioperatively has the risk of unwanted sedation. Considering that patients who undergo cardiac surgery are usually elderly (>60 yrs), as also observed in our study, high or multiple doses of pregabalin were excluded as an option since this population is more sensitive to those possible negative effects [27]. That was the main reason why we preferred such low doses of pregabalin to be given to the patients included in our study. Extra corporeal circulation during cardiac surgery is also linked with high-risk complications [17] after surgery, such as stroke, embolism, delusion-dementia, etc., and for this reason too, we did not want surgery-related complications to be mistaken as pregabalin side effects and vice versa, since the level of consciousness after major cardiac surgery is very important and is associated with better outcomes [28].

Other studies have reported that low dosages of pregabalin administered perioperatively reduce acute postoperative pain, which may help prevent the transition to chronic postsurgical pain [29,30]. It is well known that among the factors that contribute to the development of persistent postsurgical pain is the intensity of acute postsurgical pain after surgery [21]. Poorly managed pain in the early postoperative period has been linked with the high prevalence of chronic postoperative pain. By administering a single dose of pregabalin 150 mg one hour before total knee arthroplasty, J.K. Lee et al. recorded significantly lower patient pain scores during the early postoperative hours and consumed less PCA with fentanyl than in patients in the control group [30]. There are limitations to this study, as it was carried out at a single center with a relatively small number of patients, but the results have great clinical interest in that they strengthen the presumption that even a single dose of pregabalin in low dosages, when administered preoperatively, can help reduce acute postoperative pain. The analgesic result is attributed to the effect of pregabalin on postoperative hyperalgesia and central sensitization. Future studies would benefit from a larger number of patients and the use of more detailed recording of sleep disturbances with the use of special questionaries.

## 5. Conclusions

Based on the results of this exploratory study, it seems that the oral administration of a single preoperative dose of pregabalin may be associated with a significant preventive effect on chronic persistent postoperative pain after elective cardiac surgery.

## Figures and Tables

**Table 1:** Demographic and clinical patient characteristics. Data reported as mean ± SD, median (range), or number of patients, as appropriate. This is part of the table presented in our earlier study [11], reproduced to show the characteristics of the patient sample group. No statistical differences between these patients were found concerning variables presented here, comorbidities or prescribed medication [11].

Variable	Group 1 (Control) *N* = 31	Group 2 (Pregabalin 75 mg) *N* = 31	Group 3 (Pregabalin 150 mg) *N* = 31	*p*
Age (years)	66.1 ± 10.2	67.4 ± 7.8	67.8 ± 6.8	0.705
Sex (M/F) n	21/10	19/12	17/14	0.58
Weight (kg)	82.2 ± 14.5	80.3 ± 12.0	79.6 ± 8.9	0.665
Height (m)	1.70 ± 0.07	1.68 ± 0.07	1.67 ± 0.07	0.342
BMI (kg/m^2^)	28.6 ± 4.8	28.4 ± 3.9	28.6 ± 3.2	0.973
ASA status	3 (3, 4)	3 (3, 4)	3 (3, 4)	0.109

**Table 2:** Groups and results for postoperative analgesic use (No/Yes), pain intensity (NRS), and sleep disturbances (No/Yes) 12 months and 24 months after cardiac surgery.

Variable	Group 1 (Control) N = 31	Group 2 (Lyrica 75 mg) N = 31	Group 3 (Lyrica 150 mg) N = 31	*p*
NRS ^1^ 12 months (SD)	1.71 (0.588)	1.23 (0.560)	1.19 (0.402)	0.001
NRS 24 months (SD)	1.19 (0.543)	0.84 (0.454)	0.84 (0.374)	0.005
Analgesics use 12 months	16	20	6	0.027
Analgesics use 24 months	9	3	1	0.01
Sleep Disturb 12 months	8	4	1	0.037
Sleep Disturb 24 months	6	2	1	0.076

^1^ **NRS** is expressed as every group’s mean value. The remaining numbers in this table represent the number of patients who made use of analgesics or had sleep disturbances during certain time periods (12 or 24 months) after surgery. N: is the number of patients in each group.

**Table 3:** Post hoc comparisons between groups. Groups were compared using Mann–Whitney test.

Groups Compared	NRS 12 Months *p*	NRS 24 Months *p*	Analgesics 12 Months *p*	Analgesics 24 Months *p*	Sleep Disturbances 12 Months *p*	Sleep Disturbances 24 Months *p*
1 versus 2 (control versus pregabalin 75 mg)	0.009	0.024	ns	ns	ns	ns
1 versus 3 (control versus pregabalin 150 mg)	0.003	0.015	0.024	0.018	ns	ns
2 versus 3 (pregabalin 75 mg versus pregabalin 150 mg)	ns	ns	ns	ns	ns	ns

ns: non significant.

## Data Availability

Data can be found at ClinicalTrials.gov identifier: NCT01701921.

## Abbreviations

The following abbreviations are used in this manuscript:

CPSP	Chronic Postsurgical Pain
IASP	International Association of the Study of Pain
CABG	Coronary Artery Bypass Grafting
PCA	Patient Controlled Analgesia
